# LPS-Induced Endotoxemia Evokes Epigenetic Alterations in Mitochondrial DNA That Impacts Inflammatory Response

**DOI:** 10.3390/cells9102282

**Published:** 2020-10-13

**Authors:** Björn Koos, Eva Lotta Moderegger, Katharina Rump, Hartmuth Nowak, Katrin Willemsen, Caroline Holtkamp, Patrick Thon, Michael Adamzik, Tim Rahmel

**Affiliations:** Klinik für Anästhesiologie, Intensivmedizin und Schmerztherapie, Universitätsklinikum der Ruhr Universität Bochum Knappschaftskrankenhaus Bochum, 44892 Bochum, Germany; bjoern.koos@rub.de (B.K.); elmoderegger@gmail.com (E.L.M.); katharina.k.rump@rub.de (K.R.); hartmuth.nowak@kk-bochum.de (H.N.); KatrinMaria.Willemsen@kk-bochum.de (K.W.); Caroline.Holtkamp@gmx.de (C.H.); Patrick.Thon93@gmail.com (P.T.); michael.adamzik@kk-bochum.de (M.A.)

**Keywords:** sepsis, mitochondrial DNA, methylation

## Abstract

Mitochondrial DNA (mtDNA) plays a vital role as a damage-associated molecular pattern in sepsis being able to shape the immune response. Since pathogen recognition receptors of innate immune cells are activated by demethylated DNA only, we set out to investigate the amount of DNA methyltransferase 1 (DNMT1) in mitochondria and the extent of mtDNA methylation in a human endotoxin model. Peripheral blood mononuclear cells of 20 healthy individuals were isolated from whole blood and stimulated with lipopolysaccharide (LPS) for 48 h. Subsequently, DNMT1 protein abundance was assessed in whole cells and a mitochondrial fraction. At the same time, methylation levels of mtDNA were quantified, and cytokine expression in the supernatant was measured. Despite increased cellular expression of DNMT1 after LPS stimulation, the degree of mtDNA methylation slightly decreased. Strikingly the mitochondrial protein abundance of DNMT1 was reduced by 50% in line with the lower degree of mtDNA methylation. Although only modest alterations were seen in the degree of mtDNA methylation, these strongly correlated with IL-6 and IL-10 expression. Our data may hint at a protein import problem for DNMT1 into the mitochondria under LPS stimulation and suggest a role of demethylated mtDNA in the regulation of the inflammatory immune response.

## 1. Introduction

Sepsis is a highly complex immunological syndrome and one of the leading causes of death world-wide, affecting millions each year. In appreciation of our growing pathophysiological understanding, sepsis was redefined in 2016 as an acute organ dysfunction caused by a dysregulated immune response [1]. How and why the immune system is dysregulated is still under debate. The initial inflammatory response towards the invading pathogen is followed or even contemporarily accompanied by an overwhelming anti-inflammatory reaction [2]. The improper interaction between inflammation and anti-inflammation contributing to a harmful immune reaction may represent a key element in sepsis pathology, but clear insights explaining this imbalance are still elusive. Damage-associated molecular patterns (DAMPs) seem to play a major role in this maladaptive regulation [3,4]. Especially mitochondrial DNA (mtDNA), not least because of its bacterial origin, is discussed as potent DAMP able to aggravate inflammatory response [5,6]. However, it has also been described with strong anti-inflammatory patterns [7], which indicates a potential key role of mtDNA in the dysregulated immune response in sepsis. Very similar to bacterial DNA and in contrast to nuclear DNA (nucDNA), mtDNA is able to activate Toll-like receptor 9 (TLR-9) signaling [6]. Among other factors, this results from a higher frequency of CpG dinucleotides and a lower degree of methylation in prokaryotic and mtDNA [8,9]. Thus, methylation of CpG islands is one important way of how the cell prevents nuclear DNA (nucDNA) from TLR-9 binding and from being immunologically active [9].

Interestingly many reports showed an increase in the expression of DNA methylating enzymes such as DNA methyltransferase 1 (DNMT1) in septic patients and in endotoxin cell culture models of sepsis [10,11]. DNMT1 is a well-known factor for maintenance of the methylation pattern in the cell [12] and one of the few methyltransferases that also acts on mtDNA [13]. This suggests that mtDNA might also be hyper-methylated in sepsis or under endotoxic conditions, which could serve as a protective mechanism to attenuate TLR-9 activation. However, data regarding this important topic is lacking. Therefore, we tested the hypothesis whether increased DNMT1 expression could act as a compensatory factor, increasing mtDNA methylation in order to limit its immunological activity.

## 2. Materials and Methods

### 2.1. Study Design and Oversight

We conducted this prospective, in-vitro study covering blood sampling of twenty healthy volunteers. The Ethics Committee of the Medical Faculty of the Ruhr-University of Bochum (protocol no. # 17-6154) reviewed and approved this study that was also registered in the German clinical trial database (DRKS00012965). Written informed consent was obtained from all twenty healthy volunteers. This study adheres to the Declaration of Helsinki, good clinical practice guidelines, and local regulatory requirements.

### 2.2. Volunteer Recruitment and Cell Culture

We enrolled 20 healthy volunteers that were free of infections for at least four weeks prior to recruitment. Up to 70 mL of blood was drawn after informed consent and directly processed. We isolated peripheral blood mononuclear cells (PBMCs) using a density gradient centrifugation protocol (Ficoll Paque solution, GE Healthcare Bio Science AB, Uppsala Sweden). Isolated cells were resuspended in full RPMI 1640 medium (Invitrogen, Carlsbad, CA, USA) containing 10% fetal calf serum (FCS) (Biochrom AG, Berlin, Germany) and 100 U/mL penicillin plus 100 μg/mL streptomycin (both Invitrogen) and held at 37 °C in a humidified atmosphere containing 5% CO_2_. For stimulation with lipopolysaccharide (LPS), cells were seeded into a 24-well plate. With the exception of control cells, all wells were stimulated with 1 μg/mL LPS for multiple periods of time (0 h/control, 0.5 h, 2 h, 4 h, 6 h, 24 h and 48 h).

### 2.3. Enzyme-Linked Immunosorbent Assay

ELISA analysis was performed for the characterization of the immune response upon LPS stimulation. Supernatant of cells incubated with LPS (time points as indicated above) was used for the quantification of IL-6, TNF-α, and IL-10 using the Legend Max ELISA kits (BioLegend, San Diego, CA, USA). By using a calibration series, a concentration in pg/mL for each cytokine could be derived.

### 2.4. Isolation of Mitochondria

Mitochondria of PBMCs were isolated, as described previously [14]. Briefly, the protocol involves osmotic swelling and shredding of the cells to release the mitochondria. The isolated mitochondria are then separated from the cytosol as well as the cellular debris. The pure mitochondria are then lysed, and protein is isolated.

### 2.5. Western Blot

For relative quantification of DNMT1 western blot analysis was performed. Whole-cell lysates and mitochondrial lysates were used for 0 h (control) and 48 h of LPS incubation. SDS-polyacrylamide electrophoresis was performed using the 4–20% Criterion TGX Stain-Free Protein gels (Bio Rad Laboratories, Hercules, CA, USA). The transferal of the separated proteins onto a nitrocellulose membrane was confirmed by Ponceau-S staining. After blocking, primary antibodies were incubated overnight (DNMT1, 1:100, ab13537, Abcam; TRAP1, 1:500, HPA044227, Sigma Aldrich, St. Louis, MO, USA; Actin 1:10 000, MAB1501R, Sigma Aldrich). Upon rigorous washing, secondary antibodies were added (anti-mouse: 1:15 000, 115-035-207, Jackson Immuno Research, West Grove, PA; and anti-rabbit, 1:15 000, 111-035-144, Jackson Immuno Research). Visualization was done using the Clarity Western ECL Substrate (Bio-Rad). Bands were quantified using FIJI software [15] with normalization for mitochondrial DNMT1 by TRAP1 expression and for cellular DNMT1 by actin expression.

### 2.6. Methylation Analysis

Cells were lysed at each time point, and DNA was extracted using QIAamp Blood DNA kit (Qiagen, Hilden, Germany) according to the manufacturer’s instructions. DNA concentration was measured, and samples frozen at·−80 °C until used. Methylation was analyzed using the EpiJET DNA Methylation Analysis kit (Thermo Fisher Scientific, Waltham, MA, USA) according to the manufacturer’s instructions. Briefly, each DNA sample was split into three different reactions. The first served as the control, while the second and third were incubated with HpaII and MspI, respectively. After the inactivation of the restriction enzymes, the residual amount of target DNA was assessed via quantitative PCR.

### 2.7. Quantitative Polymerase Chain Reaction

The extent of the residual DNA was quantified using qPCR. For each of the three CpG-rich regions a specific primer pair was designed (Table 1). All primer pairs were ordered from Integrated DNA Technologies (IDT, Coralville, IA). The qPCR was performed using the GoTaq qPCR Master Mix (Promega Corporation, Madison, WI, USA) according to manufacturer’s instructions. Using the threshold cycle values for the undigested (*U*) and the HpaII-digested (*H*), we could calculate the % methylation as $m\%=\frac{100}{2^{H-U}}$. The MspI-digested sample served as a control for complete digestion.

The extent of mtDNA copies was also assessed by qPCR. For this a primer pair corresponding to the *mtND1* region on the mitochondrial genome was designed. To normalize mtDNA copies to nuclear DNA, primer targeting the DNA sequence of the nuclear coded *18S-rRNA* gene was used. qPCR was performed using GoTaq qPCR Master Mix (Promega) according to manufacturer’s instructions.

### 2.8. Statistical Analysis

Statistical analysis was performed using SPSS (version 25, IBM, Chicago, IL, USA). All tests were conducted with an a priori two-sided alpha error of 0.05. For all statistical testing to evaluate differences between two groups (usually LPS stimulation vs. nonstimulated control), the Wilcoxon test for nonparametrical, connected data was employed. For correlation analysis, the bivariate Spearman correlation was used.

## 3. Results

### 3.1. Characterisation of the Immune Response

**Figure 1:**
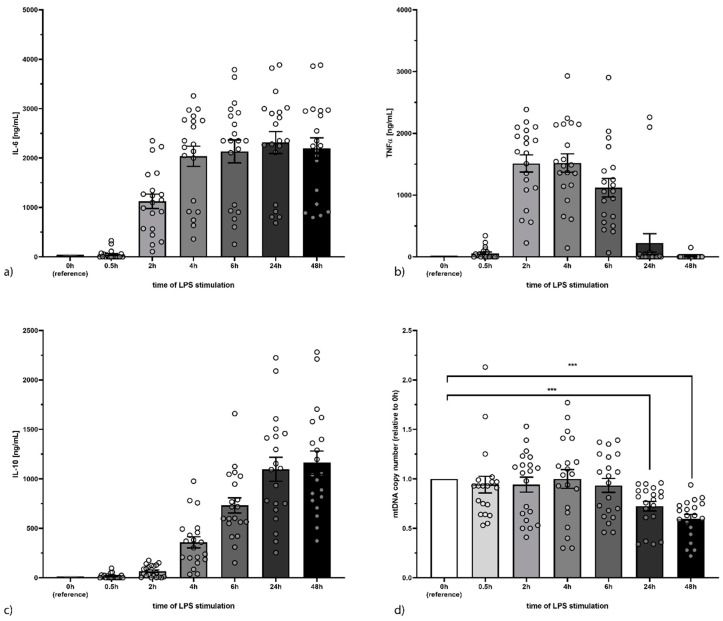
Secretion of IL-6 (**a**), TNF-α (**b**), and IL-10 (**c**) increased upon LPS stimulation. IL-6 and IL-10 did so in a sustained manner over 48 h while TNF-α only showed a transient activation. The mitochondrial DNA copy number (**d**) decreased after 24 h and 48 h of LPS stimulation (***: *p* < 0.001).

For this study 20 healthy volunteers were recruited (mean age 31.1 years ± 8.6, 11 female and 9 male). Upon stimulation of PBMCs from these individuals with LPS, we could observe a sustained and multiple increases of IL-6 concentration in the supernatant reaching more than 2000 pg/mL at 4 h (Figure 1a, *p* < 0.001). The concentration of TNF-α increased significantly already after 30 min of stimulation with LPS (60 ± 92 pg/mL; *p* = 0.002), showing a transient activation curve with a high between 2 h and 4 h (1512 ± 620 pg/mL and 1519 ± 664 pg/mL, respectively) and levels dropping back to control values after 24 h and 48 h (Figure 1b). IL-10 concentration showed a delayed increase compared to TNF-α and IL-6, reaching significance after 2 h (66 ± 60pg/mL; *p* < 0.001) while showing a sustained response with peak values at 24 h (1097 ± 539 pg/mL) and 48 h (1164 ± 522 pg/mL; both *p* < 0.001; Figure 1c). Mitochondrial DNA (mtDNA) copy number relative to genomic DNA content declined after 24 h (71 ± 22% of initial value; *p* < 0.001) and 48 h (60 ± 19% of initial value; *p* < 0.001) suggesting a failure of the mitochondria to function properly, a common hallmark in sepsis (Figure 1d).

### 3.2. Mitochondrial Concentration of DNA Methyltransferase 1

**Figure 2:**
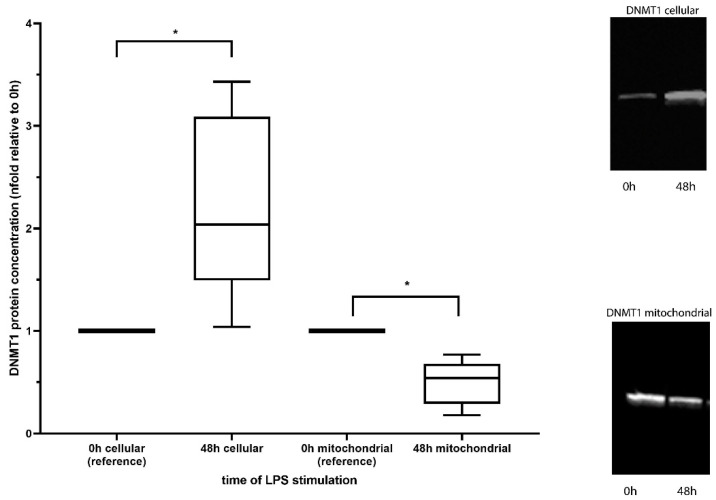
DNMT1 protein measurement. Although cellular expression of DNMT1 increased after LPS stimulation, the amount of this protein reaching the mitochondria decreased (*: *p* < 0.05).

Cellular expression as well as mitochondrial concentration of DNMT1 was assessed by Western blot analysis in 5 volunteers. We could show an increase of DNMT1 expression (cellular) after 48 h of incubation with LPS (2.24-fold ± 0.9, *p* = 0.043), while the amount of DNMT1 reaching the mitochondria dropped significantly over the same time period (0.49-fold ± 0.22, *p* = 0.043, Figure 2).

### 3.3. Identification of Methylated Regions in the Mitochondrial Genome

**Figure 3:**
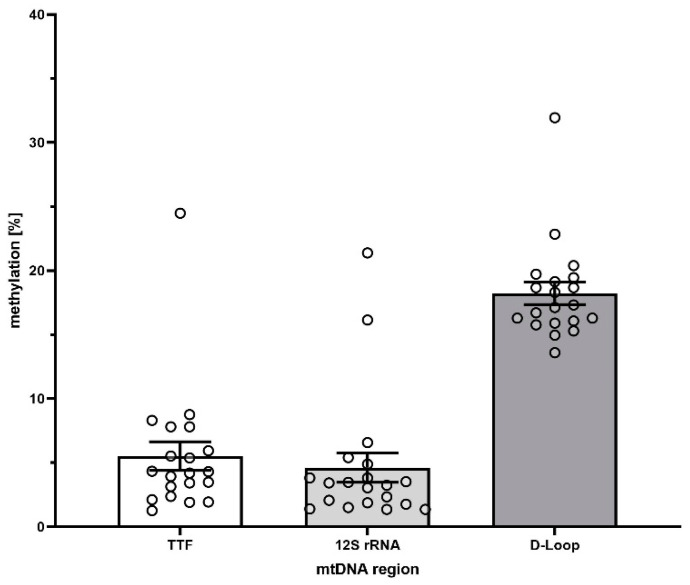
Methylation levels of three different regions of the mtDNA only showed the *D-Loop* region to be significantly methylated.

We evaluated three different CpG rich regions in the mitochondrial genome with specific primers in order to identify highly methylated CpG islands at basal conditions (Figure 3). While the CpG island near the *12S-rRNA* as well as the region corresponding to *TTF* only show very low levels of methylation (4.6 ± 4.8% and 5.7 ± 4.9%, respectively), only the CpG island near the *D-Loop* region of the mitochondrial genome showed promising results (18.3 ± 3.9%). In subsequent analyses, we focused on the *D-Loop* region. All data regarding the *12S-rRNA*, as well as the *TTF* region, are provided as Supplemental Material (Figure S1).

### 3.4. D-Loop DNA Methylation after LPS Stimulation

**Figure 4:**
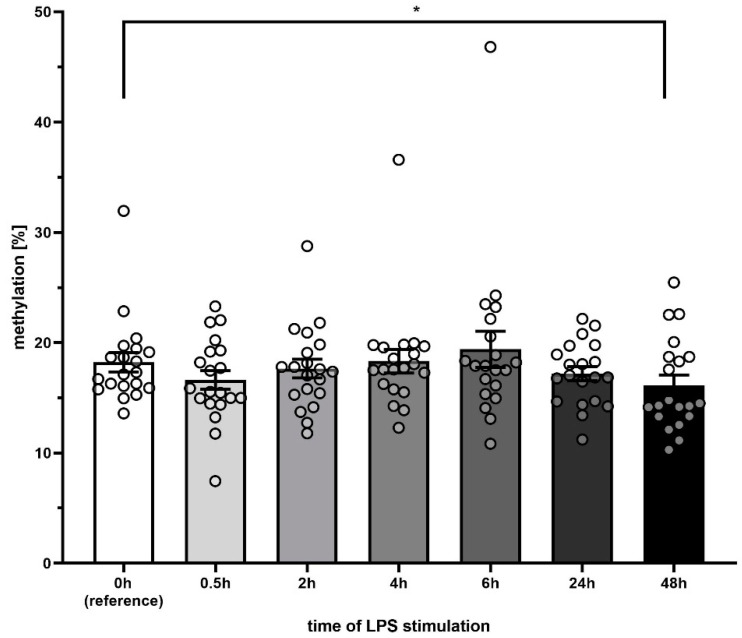
Methylation levels of the *D-Loop* region decreased upon LPS stimulation, slightly (*: *p* < 0.05).

The methylation in the CpG island near the *D-Loop* region of the mitochondrial DNA did not decrease significantly for 2, 4, 6 or 24 h, only reaching statistical significance after 48 h of incubation (15.8 ± 3.9% vs. 18.3 ± 3.9% at 0 h, *p* < 0.001). Although significant, the difference was only marginal (Figure 4). A closer analysis of the individual values reveals a biphasic distribution along with statistically significant differences at 48 h; this calls into question whether a decrease in methylation of 2–5% can really confer a biological or clinical impact.

### 3.5. Mitochondrial DNA Methylation Correlates with Cytokine Production

**Figure 5:**
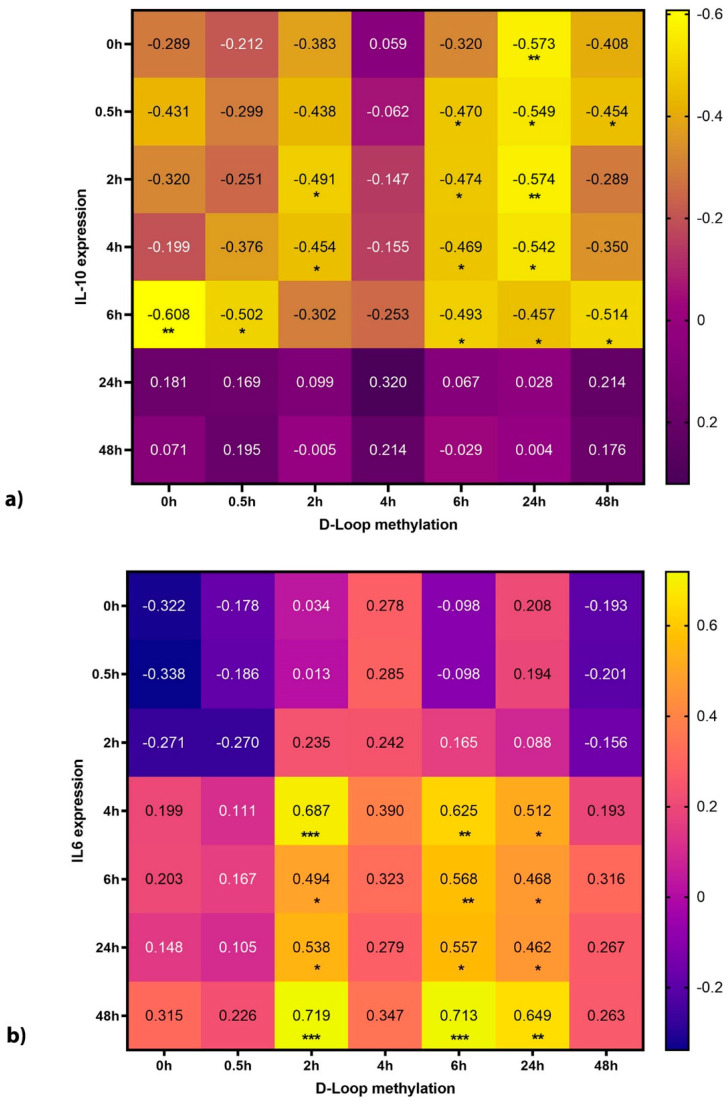
Correlation matrix of *D-Loop* methylation with IL-10 (**a**) and IL-6 (**b**). (**a**) Early (up to 6 h) IL-10 secretion correlates negatively with *D-Loop* methylation. Especially the early *D-Loop* methylation correlates well with 6 h of IL-10 secretion. In addition, later *D-Loop* methylation levels (6 h and 24 h) correlate well with early (up to 6 h) IL-10 values. (**b**) Correlation between IL-6 and *D-Loop* methylation is mostly between later time points. In general, earlier *D-Loop* methylation time points correlate better with later IL-6 time points. (Numbers depict correlation coefficient and *: *p* < 0.05, **: *p* < 0.01, ***: *p* < 0.001).

In order to elucidate the potential biological role of our findings, we correlated the extent of methylation in the *D-Loop* region after incubation with LPS with the cytokine concentration at different time points. The extent of methylation correlated well with the concentration of IL-6, a key pro-inflammatory cytokine (Figure 5), as well as IL-10, a key cytokine of the anti-inflammation. A correlation with TNF-α could not be observed.

## 4. Discussion

### 4.1. DNMT1 Abundance Is Reduced in Mitochondria in an Endotoxin Cell Culture Model

In our work, we could verify that PBMCs in an endotoxemia model of sepsis showed an increased cellular expression of the methyltransferase DNMT1 [10,16]. Critically, at the same time, the levels of DNMT1 reaching the mitochondria were distinctly reduced. This is in line with our recent study showing that the mitochondrial transcription factor A (TFAM), which is also upregulated after LPS treatment suffers from the same dilemma, namely not reaching the mitochondria [17]. Taken together, our results hint at an insufficient translocation of mitochondrial proteins that need to be shuttled from the cytoplasm into the mitochondrial matrix [18]. These results, therefore, underline that when studying mitochondria, the protein abundance within the mitochondria needs to be considered in addition to their cellular expression. While a mechanistic evaluation of this phenomenon might be worthwhile, it certainly is beyond the scope of this study. Given that the function of DNMT1 is thought to be maintaining the methylation pattern [19] a reduced mitochondrial abundance of this enzyme could lead to a reduced mtDNA methylation.

### 4.2. Methylation of mtDNA Is only Marginally Reduced upon LPS Treatment

In order to investigate this hypothesis, we evaluated three different CpG-rich regions of the mitochondrial DNA regarding their degree of methylation. In order to avoid artifacts introduced by incomplete bisulfide conversion, which affects methylation analyses of mtDNA regularly [20], we chose a restriction enzyme-mediated approach that does not suffer from this limitation. According to the literature [20,21] we found that most regions of the mtDNA are basically only very marginally methylated. The CpG island in the *D-Loop* region, however, showed a robust methylation of 18.3%. Therefore, we chose this CpG island for further analysis. Interestingly, in the time series experiments, we found that while the abundance of DNMT1 protein changed quite extensively upon LPS treatment, the methylation of the *D-Loop* region only reduced marginally. This is in stark contrast to the finding that nuclear DNA is often hyper-methylated during sepsis and in endotoxin models [22], highlighting again the difference between nucDNA and mtDNA. Therefore, DNA released from the mitochondrion could be capable to act as an agonist for TLR-9 and subsequent signaling. This is in line with the finding, that free circulating mtDNA correlates with the survival in sepsis patients [23]. However, whether such a small methylation difference, despite being statistically significant, could have a biological or even a clinical impact is questionable. But can we simply dismiss the possibility of a biological relevance?

### 4.3. D-Loop Methylation Levels Correlate Strongly with Cytokine Release

In order to explore a potential impact associated with the small alterations in the degree of mtDNA methylation, we performed a correlation analysis between *D-Loop* methylation and secretion of the inflammatory cytokines TNF-α, IL-6 and IL-10. To our own surprise, we could indeed observe a strong correlation between the methylation levels and the concentration of IL-6 and IL-10 but not TNF-α in the supernatant of the cells. This was startling, given the low methylation changes upon LPS stimulation. Furthermore, the correlation with IL-6 was positive, indicating higher IL-6 expression with higher methylation levels. In contrast to these results, Timmermann et al., did not find a correlation between mtDNA concentration in plasma with IL-6 concentrations [24]. However, they studied peak levels of mtDNA without assessing the degree of methylation, which is fundamentally different from our approach. Interestingly we could also observe a negative correlation between mtDNA methylation levels with IL-10, indicating an association between lower methylation with higher IL-10 expression. In contrast to IL-6, IL-10 is typically associated with the anti-inflammatory response, linking mtDNA to this process. This is in line with Schäfer et al. [7] linking mtDNA to immune paralysis. Interestingly, while the negative correlation of IL-10 with the *D-Loop* methylation is strong at early time points of the methylation analysis, it diminishes until 4 h and then increases again, leading to an oscillation effect (from left to right in Figure 5a). The same can be observed in the correlation between IL-6 and *D-Loop*. Here the correlation increases until 2 h (on the *x*-axis), then drops at 4 h and increases again. More experiments are needed to explore this interesting mechanism. Furthermore, it has to be pointed out that the association between methylation and cytokine secretion is only statistical in nature. However, it is intriguing to speculate about the mechanisms involved. It is theoretically possible that this statistical association might be directly linked to the de-methylation of the *D-Loop* region. Cytokine expression could for example, be due to the activation of TLR-9 or other receptors. Another explanation could be a global de-methylation of mitochondrial DNA. This global change could also include regions with a higher change of methylation levels, which, therefore might be the causative agents (again via toll like receptors). A possible explanation for this global change is the diminished levels of DNMT1 in mitochondria. DNMT1 is responsible for methylation maintenance and a lack of this enzyme can lead to lower overall methylation levels across the entire mitochondrial genome. Of course, our results are just of an associative nature, thus further work will be needed in order to explore this phenomenon.

## 5. Conclusions

In conclusion, we found that cellular expression of DNMT1 increased upon LPS stimulation. In contrast to this, the mitochondrial abundance of this methylation maintenance enzyme decreased upon the same treatment, suggesting an import failure. Furthermore, while methylation effects on mtDNA, probably mediated by lack of DNMT1, are only very small—they are strongly associated with IL-6 and IL-10. This hints at a relevance of mtDNA methylation in maintenance of the perpetual immune response. Further work is needed to fully investigate and explain this phenomenon.

## Figures and Tables

**Table 1:** Primer sequences for methylation analysis.

mtDNA Region	Forward Primer	Reverse Primer
*D-Loop*	CTCGTCCCCATGGATGACCC	TGAAGTAGGAACCAGATGTCGGA
*TTF*	CACCCAAGAACAGGGTTTGT	TGGCCATGGGTATGTTGTTAAG
*12S-rRNA*	GGTCACACGATTAACCCAAGT	TGTTAAAGCCACTTTCGTAGTCTAT
*mtND1*	CACCCAAGAACAGGGTTTGT	TGGCCATGGGTATGTTGTTAA
*18S-rRNA*	TAGAGGGACAAGTGGCGTTC	CGCTGAGCCAGTCAGTGT

## Supplementary Materials

The following are available online at https://www.mdpi.com/2073-4409/9/10/2282/s1, Figure S1: Course of methylation change of *TTF* and 12S-RNA upon LPS stimulation.
