# Supplementary material for: LPS-Induced Endotoxemia Evokes Epigenetic Alterations in Mitochondrial DNA That Impacts Inflammatory Response
Source: Cells. 2020 Oct 13;9(10):2282. doi: 10.3390/cells9102282 (PMC7650703; doi:10.3390/cells9102282)
Supplement: Supplementary file 1 [file cells-09-02282-s001.zip › SupplFigLegends.docx]

Supplementary Figure S1: Course of methylation of the TTF region (a) and the 12S-rRNA region (b) of the mitochondrial genome upon LPS stimulation.

Supplementary Figure S2: Western Blot of VDAC2 protein expression (red arrow) in the cytoplasm (odd numbers) and mitochondria fraction (even numbers).
