# Supplementary figures and images for: LPS-Induced Endotoxemia Evokes Epigenetic Alterations in Mitochondrial DNA That Impacts Inflammatory Response
Source: Cells. 2020 Oct 13;9(10):2282. doi: 10.3390/cells9102282 (PMC7650703; doi:10.3390/cells9102282)

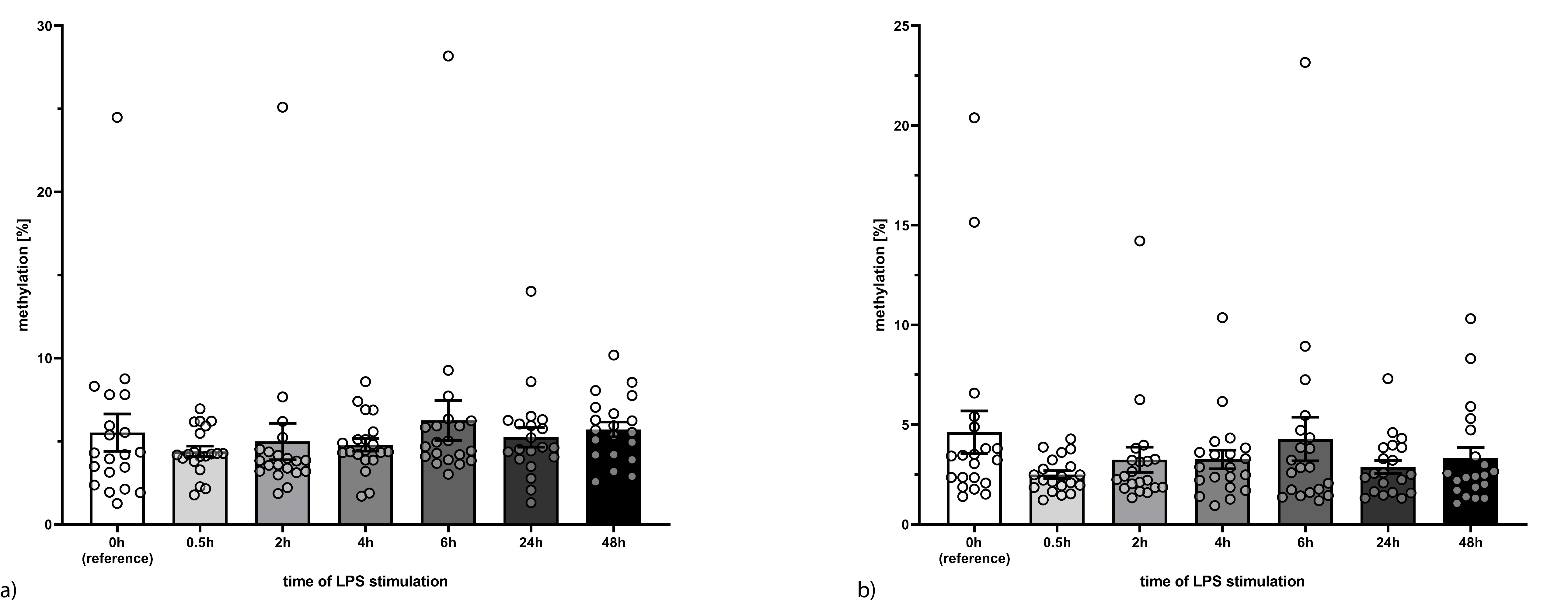

Supplement: Supplementary file 1 [file cells-09-02282-s001.zip › Suppl Fig 1.jpg]

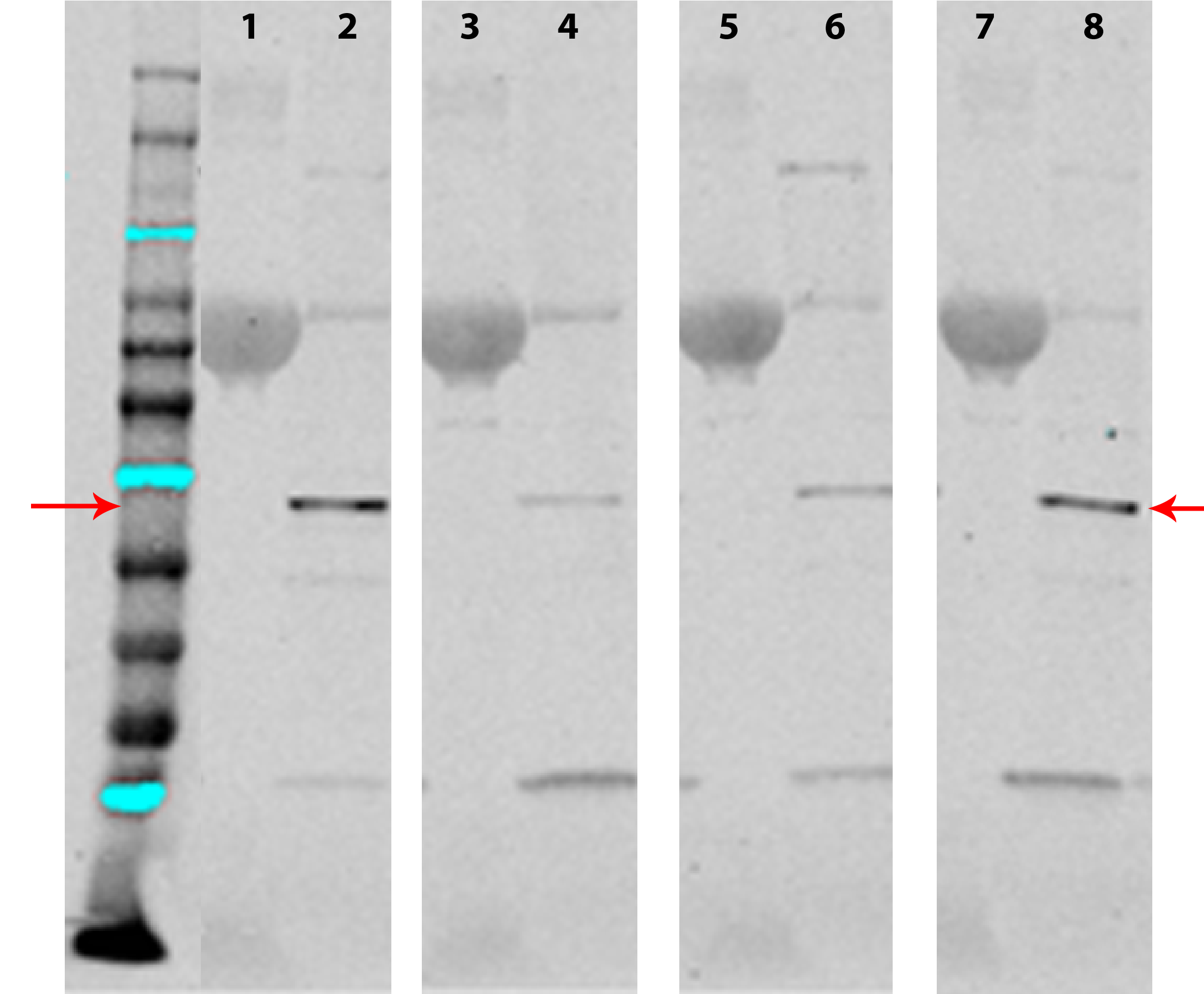

Supplement: Supplementary file 1 [file cells-09-02282-s001.zip › Suppl Fig 2.png]
